# Application of Nitrogen and Carbon Stable Isotopes (δ^15^N and δ^13^C) to Quantify Food Chain Length and Trophic Structure

**DOI:** 10.1371/journal.pone.0093281

**Published:** 2014-03-27

**Authors:** Matthew J. Perkins, Robbie A. McDonald, F. J. Frank van Veen, Simon D. Kelly, Gareth Rees, Stuart Bearhop

**Affiliations:** 1 Centre for Ecology and Conservation, University of Exeter, Penryn, Cornwall, United Kingdom; 2 Environment and Sustainability Institute, University of Exeter, Penryn, Cornwall, United Kingdom; 3 Food and Environment Research Agency, York, Yorkshire, United Kingdom; Behavioural Ecology & Ecophysiology group, Denmark

## Abstract

Increasingly, stable isotope ratios of nitrogen (δ^15^N) and carbon (δ^13^C) are used to quantify trophic structure, though relatively few studies have tested accuracy of isotopic structural measures. For laboratory-raised and wild-collected plant-invertebrate food chains spanning four trophic levels we estimated nitrogen range (NR) using δ^15^N, and carbon range (CR) using δ^13^C, which are used to quantify food chain length and breadth of trophic resources respectively. Across a range of known food chain lengths we examined how NR and CR changed within and between food chains. Our isotopic estimates of structure are robust because they were calculated using resampling procedures that propagate variance in sample means through to quantified uncertainty in final estimates. To identify origins of uncertainty in estimates of NR and CR, we additionally examined variation in discrimination (which is change in δ^15^N or δ^13^C from source to consumer) between trophic levels and among food chains. δ^15^N discrimination showed significant enrichment, while variation in enrichment was species and system specific, ranged broadly (1.4‰ to 3.3‰), and importantly, propagated variation to subsequent estimates of NR. However, NR proved robust to such variation and distinguished food chain length well, though some overlap between longer food chains infers a need for awareness of such limitations. δ^13^C discrimination was inconsistent; generally no change or small significant enrichment was observed. Consequently, estimates of CR changed little with increasing food chain length, showing the potential utility of δ^13^C as a tracer of energy pathways. This study serves as a robust test of isotopic quantification of food chain structure, and given global estimates of aquatic food chains approximate four trophic levels while many food chains include invertebrates, our use of four trophic level plant-invertebrate food chains makes our findings relevant for a majority of ecological systems.

## Introduction

Understanding food web structure is of critical importance to a broad suite of ecological theory given that trophic dynamics between individuals, populations, species and functional guilds underpin the ecological functioning and evolution of biological communities [1], [2]. Quantifying food web structure (trophic structure hereafter) is therefore a prerequisite to better understand how it in turn interacts with emergent properties of organisms and the environment, such as energy flux [3], population dynamics [4], patterns of biodiversity [5] and ecosystem functioning [6], [7]. Determination of feeding relationships between species is integral to quantifying trophic structure, and traditional methods include gut-content analysis, faecal analysis and behavioural observations. However, these methods can be laborious and may not reflect variation in digestibility and assimilation of source items, and if limited in their collection in space and time, may lead to over or under representation of source contributions [8]. Increasingly, stable isotope ratios of nitrogen (N^15^:N^14^, termed δ^15^N) and carbon (C^13^:C^12^, termed δ^13^C) in consumer tissues are used to provide a temporally and spatially integrated construct of dietary niche [8], with δ^15^N and δ^13^C of consumer proteins reflecting the proteins of their food sources [9], [10]. Typically, enrichment in δ^15^N of 2.5‰ to 3.4‰ is observed from diet to consumer [11]–[13], allowing determination of an organism's trophic level [11], [14], [15] and overall food chain length [16], [17]. Conversely, enrichment in δ^13^C is much smaller between diet and consumer [11], [13], and because basal sources often differ in their δ^13^C values, δ^13^C can be used to trace prey–consumer connections or food chains [11]. Hence change in δ^15^N and δ^13^C from source to consumer as described (termed trophic discrimination factors and represented as Δδ^15^N or Δδ^13^C), is the mechanism that crucially underpins the positioning of individuals, populations and species relative to one another in bivariate isotopic space (typically with δ^15^N on a y-axis and δ^13^C on an x–axis). Importantly this subsequently allows for measures of Euclidean distances across the isotopic space occupied by populations, species or communities in order to quantify aspects of trophic structure [18]–[21]. For instance, food chain length is calculated as λ + (nitrogen range/average Δδ^15^N), where λ is minimum trophic position and nitrogen range (NR) is mean difference between trophic levels of maximum and minimum δ^15^N [11], [14], [18]. Similarly, carbon range (CR) measures breadth of trophic sources and is calculated using mean difference between maximum and minimum δ^13^C of community members [18]. Observational studies have largely used such measures to quantify food chain length, typically in aquatic systems, in response to factors such as ecosystem size, disturbance and productivity [22]–[24]. Thus the use of stable isotope ratios in an organism's tissues to provide temporally and spatially integrated dietary data is proving a very valuable methodology for trophic research.

Critically though, variation in Δδ^15^N and Δδ^13^C generates uncertainty/error in subsequent estimates of trophic structure and relationships [11], [17], [25]–[28]. Such variation in discrimination factors is well documented in the literature (−0.8‰ to 5.9‰ for δ^15^N and -2.7% to 3.4‰ for δ^13^C, excluding fluid feeders; [29]). This can be a consequence of multiple factors, including dietary protein quality, metabolic process and efficiency of protein assimilation and loss, fasting, growth rate, age, size, tissue type, sample size and sampling process, although there is considerable debate on which are most important [12], [13], [27], [30] (and references therein for all). It is the cumulative effect of all such underlying sources of variation that we observe as variation in discrimination factors between different individuals and species. Common practice often uses mean estimates of Δδ^15^N and Δδ^13^C ignoring variability around estimates; consequently, derived estimates of trophic structure and subsequent ecological conclusions may lack accuracy.

These issues associated with variance have driven recent innovations in the analysis of isotopic data that provide practitioners with tools to apply Bayesian inference to the calculation of trophic structure metrics [20]. These approaches are ideally suited to testing effects of variance as they provide population and community trophic metric estimates based on resampling of variance in mean δ^15^N and δ^13^C estimates, effectively quantifying and propagating variation in raw data as uncertainty in subsequent metric outputs, allowing for critical examination of precision in estimates of trophic structure.

Despite variation in discrimination being widely documented (e.g. [11], [13], [29]) to date relatively few studies have validated isotopic structural measures such as NR and CR against known measures of food chain length, despite repeated calls for research [21], [27]. Of such studies, variation in baseline δ^15^N has been shown to affect food chain length estimates [17], and variation in Δδ^15^N to affect trophic level/food chain estimates [11], [25], [28], all in aquatic systems. Such studies have therefore been vital in guiding our understanding and use of these techniques; however, most have used wild systems which may not accurately identify basal sources or preclude omnivory, and thus do not explicitly know/control trophic levels against which to compare isotopic measures of structure. To further improve our understanding of isotopic approaches to quantifying trophic structure, so it would be prudent to additionally test the accuracy of isotopic measures of trophic structure for multi-trophic level food chains when trophic levels are explicitly controlled and food chain lengths known, in addition to allowing for the propagation of variation in Δδ^15^N and Δδ^13^C to final isotopic trophic measures.

It is of further importance to understand effects of variation in Δδ^15^N and Δδ^13^C upon structural measures such as NR and CR given these univariate measures underpin other bivariate (δ^15^N with δ^13^C) measures of trophic structure in isotopic space. NR is also the most used isotopic metric in observational studies [21], and its function as a tool to quantify trophic level, food chain length or as a component of bivariate measures is dependent upon an assumed constant δ^15^N enrichment with each consumer level. Variance around this assumed average enrichment constitutes unknown error in estimates of NR in observational studies. Thus, experimental validation would improve understanding of the importance of variance in Δδ^15^N and Δδ^13^C for affecting quantifications of trophic structure in wild systems [21], strengthening subsequently derived ecological conclusions, in addition to further catalysing the development and use of these techniques to/by a wider audience of ecologists.

In this study, we use natural plant and insect food chains raised under controlled conditions to examine the dynamics of δ^15^N and δ^13^C over four trophic levels, and using resampling procedures to allow propagation of variance in Δδ^15^N and Δδ^13^C, test the accuracy of the isotopic metrics NR and CR to quantify trophic structure. We use a terrestrial plant-insect system in this study simply because terrestrial systems are currently less well studied relative to aquatic systems and it would therefore broaden our knowledge of isotopic techniques to have a greater range of validation studies to draw upon. By using four trophic levels, we broaden cross–system applicability of our results to a larger repository of aquatic studies, given that global aquatic food chain lengths have been estimated at 3.5 to 4.0 trophic levels [17]. Specifically, we test three questions: 1) How consistent are Δδ^15^N and Δδ^13^C between trophic levels and among food chains? 2) Does NR accurately determine food chain length? 3) How does CR change with food chain length?

## Methods

### Ethics Statement

All wild animals used in this experiment are not protected by law, and were collected on land belonging to the University of Exeter for which permissions to collect had been granted.

### Experimental Set-up

To test dynamics of δ^15^N and δ^13^C with changing trophic level, three replicate food chains were raised in the laboratory, with a further analogous food chain collected from the wild to allow comparison with wild systems. All food chains had four trophic levels consisting of: primary producer (plant) → herbivore (aphids feeding on plant phloem sap) → predator (hoverfly larvae feeding on aphids) → secondary predator (parasitoid hymenoptera which are obligate endoparasites of hoverfly pupa, with a single parasitoid emerging from a single pupa). These organisms were used as they provided us the opportunity to study natural food chains (that can be found in nature) and were easy to culture in controlled laboratory conditions so that trophic levels were explicitly known. Additionally, using natural food chains allowed us to directly contrast observations of controlled lab raised food chains with our analogous wild collected food chain in order to interpret and ensure the relevance of our laboratory findings for drawing conclusions and lessons that could inform future studies using wild systems.

For laboratory food chains, grain aphids (*Sitobion avenae*) were raised on two independent food plants; One based on a C3 photosynthetic pathway (wheat *Triticum aestivum*) and the other based on a C4 pathway (maize *Zea mays*), enabling separation of the plants on a δ^13^C axis and thus broadening the generality of any observed patterns. Plants were raised on a common source of homogenised compost and distilled water, and introduced to aphids at 20 days (wheat) or 30 days (maize). 1^st^ generation larvae of wild-caught hoverfly (*Syrphus vitripennis*) were fed either wheat or maize raised aphids or an approximate 50∶50 ratio of both. Within each treatment, 24–48 hours after hatching, a random subset of hoverfly larvae were exposed to wild caught adult female parasitoids (*Diplazon laetorius*) to allow parasitic oviposition. All plants and insects were raised under a 16∶8 light∶ dark cycle at 70% humidity. Plant leaves and aphids of all ages were collected at random and frozen (−20°C) prior to tissue preparation. Hoverfly larvae entered pupation 8–10 days after hatching and after 72 hours pupation were frozen (−20°C) for later dissection. Prior experimentation identified 72 hour pupation as suitable to provide soft pupa tissue comparable to that likely consumed by parasitoid larvae (i.e. after exuviae had formed). Parasitised hoverfly larvae were allowed to complete pupae development (19–21 days) and newly eclosed adult parasitoids were frozen (−20°C) within 12 hours without having fed.

For the wild food chain, nettle (*Urtica dioica*) leaves and nettle aphids (*Microlophium carnosum*) were collected independently and frozen (−20°C) for later preparation. Hoverfly larvae (*Syrphus vitripennis*) were collected when judged at >50% grown and laboratory raised on daily-collected wild nettle aphids until pupation, under a 16∶8 light∶dark cycle at 70% humidity. Pupation proceeded until either adult hoverflies or adult parasitoids (*Diplazon laetorius*) eclosed after 10–11 or 16–20 days respectively, and were frozen (−20°C) within 12 hours without having fed. Our four replicate food chains are hereafter termed after their plants as wheat, maize, wheat + maize (w+m) and nettle. Parasitoid hymenoptera are referred to as wasp hereafter.

### Tissue Preparation & Lipid Extraction

We used tissues for sources to best represent assimilation in consumers, as shown to be important [31]. While aphids fed on plant sap, we used whole leaf tissue given difficulties of extracting sap and because whole leaf tissue δ^15^N has been shown not to differ from sap [32]. For each food chain, following dissection, soft internal tissues of 60–80 aphids were pooled to produce a single sample. Hoverfly larvae soft tissue was obtained from pupae casing. Wasps represented end consumers and we used whole tissues. For wild hoverflies and wasps we used adult whole tissues. Individual hoverflies and wasps each provided single replicates. Sample sizes for plants, aphids and hoverflies were *n* = 15 (except nettle hoverflies, *n* = 7) while wasps were more difficult to obtain: wheat (*n* = 10), w+m (*n* = 7), nettle (*n* = 6). For maize, no wasps were obtained due to high larval mortality. All samples were dried at 45°C for >48hrs and homogenised. Subsequently, insect samples were immersed in 2∶1 chloroform: methanol solution for 50 minutes to remove free lipid, and then left to air dry. Prior experimentation showed lipid extraction to have no significant effects on δ^15^N of these insects.

### Stable Isotope Analysis

For all samples, 0.5 mg±0.05 (insect) or 3 mg±0.1 (plant) dried material was enclosed in tin capsules. Stable isotope analysis (SIA) was conducted at the Food and Environment Research Agency, York, UK. Samples were analysed for δ^15^N and δ^13^C in a Fisons EA1108 elemental analyser (Carlo Erba Instruments, Milan, Italy), coupled with an Isoprime isotope ratio mass spectrometer (GV Instruments, Manchester, UK). Stable isotope ratios are reported in delta (δ) notation where δ^15^N and δ^13^C  =  [(R_sample_/R_standard_) −1] ×1000, where R is ^15^N/^14^N or ^13^C/^12^C. Isotope ratios are expressed in per mil (‰) relative to the ratio of international reference standards (R_standard_) which are Atmospheric Nitrogen and Vienna PeeDee Belemnite (VPDB) for nitrogen and carbon respectively. Measures of standards placed throughout samples exhibited acceptable instrument reproducibility of <0.09‰ (SD) for δ^15^N and <0.18‰ (SD) for δ^13^C using collagen standard, insect whole tissue standard (cockroach; *Nauphoeta cinerea*), and sucrose C4 plant standard.

### Data Analysis

Initial analyses used two-way analysis of variance (ANOVA) to test effects of the two explanatory variables *trophic level* (levels  =  plant, aphid, hoverfly or wasp) and *food chain type* (levels  =  wheat, maize, w+m or nettle) on δ^15^N and then δ^13^C. Model simplification used backwards stepwise regression from a maximal model and ANOVA model comparisons to identify non-significant terms for elimination. Homogeneity of variances and normality of model residuals were checked in all instances. To determine where significant differences lay between levels within treatments, subsequent one-way ANOVA for each food chain type were tested with Tukey post hoc tests, for both δ^15^N and δ^13^C.

Within each food chain type, we then calculated mean (± SD) Δδ^15^N and Δδ^13^C as the difference in δ^15^N or δ^13^C between each source and its consumer by randomly pairing replicates (n = 6 to 15). To establish underlying sources of variation in Δδ^15^N, two-way ANOVA tested effects of explanatory variables *trophic link* (levels  =  plant-aphid, aphid-hoverfly, or hoverfly-wasp) and *food chain type* (levels  =  wheat and nettle). Only nettle and wheat food chain data were used as these contained all three trophic links, allowing for a balanced analysis. For explanatory variables, variation in Δδ^15^N was quantified using sums of squares in model outputs and was expressed as a proportion of the null model variance. Model simplification was used as described above. All analyses were conducted in R version 2.14.1 [33].

Nitrogen range (NR) and carbon range (CR), calculated as the mean difference between trophic levels of maximum and minimum δ^15^N or δ^13^C respectively, are quantifications of trophic structure with NR representing food chain length and CR the breadth of energy sources. For all species combinations of each food chain length within each food chain type separately, NR and CR were independently calculated using resampling of uncertainty around sample mean estimates to provide probabilistic distributions representing 50%, 75% and 95% credible intervals of mean estimates for NR and CR, using the SIBER computational code [20] in the R package SIAR [34], [35]. Additionally for NR, for each food chain length, resampled mean estimates across food chain types and species combinations were pooled to produce overall 95% credible intervals.

As established by preliminary experiments, we applied correction factors of –0.7‰ for δ^15^N and +0.4‰ for δ^13^C to hoverflies on the wild nettle food chain (which used adult tissues) to make them directly comparable to larval hoverflies on laboratory food chains.

### Data Availability

All raw data used in this study are provided as supplementary material (Dataset S1).

## Results

### How consistent are Δδ^15^N and Δδ^13^C between trophic levels and among food chains?

#### δ^15^N

**Figure 1:**
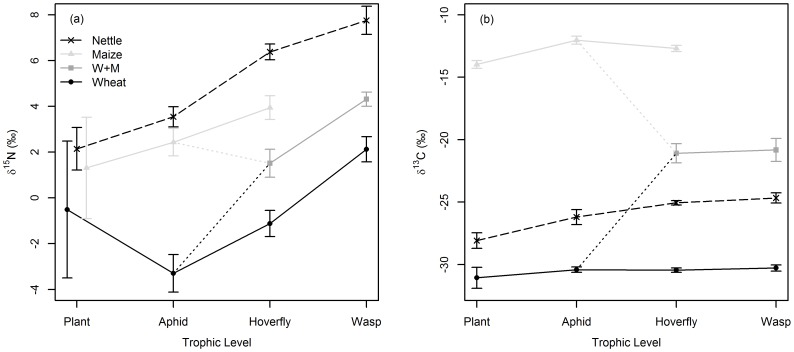
δ^15^N (a) and δ^13^C (b) across four trophic levels of four replicate terrestrial food chains. Mean ± SD (‰) are shown. *n* = 6 to 15. For plant δ^15^N, mean ± SD are offset on x-axis for clarity. Dotted lines are trophic links between two aphid prey sources and their hoverfly predator.

Though given the same nitrogen source, δ^15^N of wheat plants were depleted relative to maize plants by ≈2‰, while wild nettle plants (of independent nitrogen source) were slightly enriched (0.3‰) relative to maize. Such differences between food chain types were largely propagated to higher trophic levels (Fig. 1a) and found to be significant; two-way ANOVA showed a significant interaction between trophic level and food chain type indicating effects of these variables were interdependent (F_(6, 152)_  = 11.57, *P*<0.001). To determine if large variances in δ^15^N of maize and wheat plants (Fig. 1a) disproportionately affected these results, we repeated this analysis excluding these plants, but found no difference in outcome.

To ascertain patterns of δ^15^N discrimination, Tukey post hoc tests performed on one–way ANOVA for each food chain type established where δ^15^N differed between trophic levels. Overall, δ^15^N discrimination factors showed significant enrichment from source to consumer (range of 1.4‰ to 3.3‰), in all but three instances (Table 1). Exceptional to this trend, wheat feeding aphids showed significant average depletion in δ^15^N (−2.4‰) relative to hosts.

**Table 1:** Mean ± SD (‰) Δδ^15^N and Δδ^13^C across four trophic levels of four terrestrial food chains.

Source	→	Consumer	Food Chain			
**δ^15^N**			wheat	maize	w+m	nettle (wild)
Plant	→	Aphid	−2.40±2.63***	1.12±2.58		1.40±0.75***
Aphid	→	Hoverfly	2.18±1.25**	1.51±0.69*	4.81±0.87***(w) −0.92±1.02***(m) 1.95±3.06* (c)	3.08±0.60***
Hoverfly	→	Wasp	3.31±1.00***		2.49±0.62***	1.38±0.61**
**δ^13^C**						
Plant	→	Aphid	0.65±0.92**	1.94±0.36***		1.88±0.86***
Aphid	→	Hoverfly	−0.04±0.33	−0.69±0.42***	9.33±0.87***(w) −9.05±0.79***(m) 0.14±9.38 (c)	1.22±0.71***
Hoverfly	→	Wasp	0.20±0.30		0.39±0.65	0.40±0.31

Significant differences in δ^15^N and δ^13^C between source and consumer within each food chain indicated as *P* = <0.05*, <0.01**, <0.001***. w+m hoverfly has two aphid sources and values are given for both: (w)  =  wheat aphids, (m)  =  maize aphids, (c)  =  combined.

Given δ^15^N enrichment was broadly consistent and larger than that observed in δ^13^C (Table 1), for δ^15^N we also used two-way ANOVA to determine sources of variation in Δδ^15^N (Table 2). After disproportionate plant variation was excluded, we observed a significant interaction between trophic link and food chain type accounting for 32% of variation in Δδ^15^N. This significant interaction infers variation in Δδ^15^N as caused by these variables was species and system specific.

**Table 2:** Sources of variance in Δδ^15^N (‰).

	All combinations		Excludes primary producers	
Null model variance in Δδ^15^N	398.1		52.8	
Model Term	Proportion of null variance explained		Proportion of null variance explained	
Food Chain	0.04		0.02	
Trophic Link	0.42		<0.01	
Food Chain * Trophic Link	0.21	***	0.32	***
Full model (all terms combined)	0.67		0.34	

Variance in Δδ^15^N as accounted for by either food chain or tropic link was established by expressing ANOVA model terms as a proportion of the null model variance. Δδ^15^N is based upon differences in raw δ^15^N between trophic levels, for all source-consumer links on wheat and nettle food chains only (to provide a balanced analysis). ANOVA was conducted twice: firstly for all source-consumer links and secondly excluding all links including a primary producer. A significant interaction explained greater variance in Δδ^15^N after primary producer links were excluded. Significant model terms are indicated as *** with *P* = <0.001.

#### δ^13^C

δ^13^C values were ≈17‰ different between wheat and maize food chains, with hoverfly and wasps on the w+m food chain approximately half way between the two having integrated aphid sources from both (Fig. 1b). Two-way ANOVA showed δ^13^C was significantly affected by an interaction between trophic level and food chain type (F_(6, 152)_  = 19.22, *P*<0.001), indicating food chain effects on δ^13^C were affected inconsistently by trophic level (Fig. 1b).

Differences in δ^13^C discrimination across trophic links and food chain types were of variable direction and magnitude (−0.7‰ to 1.9‰, excluding w+m aphid-hoverfly; Table 1). Across trophic links and food chain types, Tukey post hoc analyses showed either significant enrichment (0.6‰ to 1.9‰) or no change in δ^13^C between trophic levels (Table 1).

### Does nitrogen range accurately determine food chain length?

**Figure 2:**
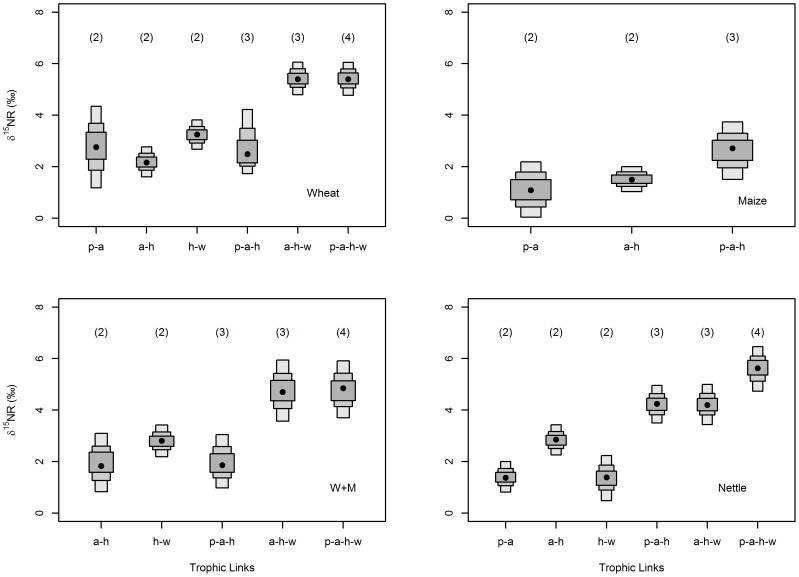
Probability distributions of mean nitrogen range (NR) for different food chain lengths for each food chain. NR is difference between mean δ^15^N (‰) of community end members, based on resampling (*n* = 10,000) of uncertainty in sample mean estimates. Black dots represent mode (of means), while shaded boxes (dark to light) show 50%, 75% and 95% credible intervals for mean estimates. No wasps were obtained for the maize food chain. x-axis labels are species identity: p = plant, a = aphid, h = hoverfly, w = wasp. Parenthesis number shows food chain length.

In most instances, estimates of nitrogen range (NR) were observed to increase with greater food chain length within each food chain (Fig. 2). Exceptional to this were low NR estimates on the wheat and w+m food chains for combinations including wheat plants.

Within a food chain, in some instances modal or credible interval values of NR varied distinctly between different species combinations of the same food chain length, but such differences were not constant across food chains suggesting a species and system specific nature to such variation. Across food chains, 95% credible interval estimates of mean NR were not generally larger than 1‰ δ^15^N either side of the modal value (Fig. 2). The exception was larger estimates on the wheat food chain when wheat plants were included as a consequence of uncertainty in mean estimates (Fig. 2), propagated from large sample variation in wheat plants (Fig. 1).

**Figure 3:**
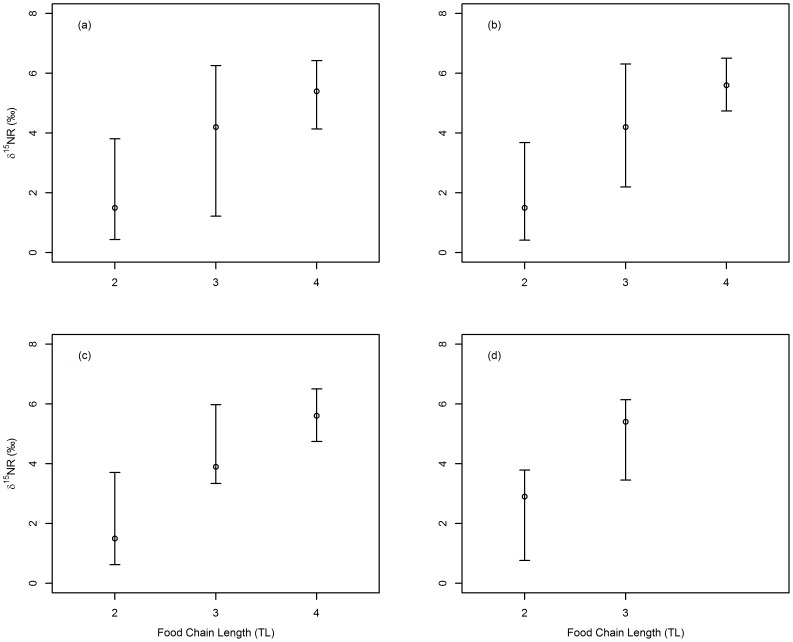
Overall nitrogen range (NR) for food chains containing two, three or four trophic levels. Values derived from combining resampled mean values from all four replicate food chains. TL  =  trophic levels. Circles represent mode (of means) and bars 95% credible intervals for mean. a) all combinations: 2 TL n = 10, 3 TL n = 7, 4 TL n = 3; b) excludes combinations that include wheat plants: 2 TL n = 9, 3 TL n = 5, 4 TL n = 1; c) excludes all combinations that include wheat or maize plants: 2 TL n = 8, 3 TL n = 4, 4 TL n = 1; d) excludes all combinations containing primary producers: 2 TL n = 7, 3 TL = 3.

Overall estimates of NR based on combining resampled mean estimates from all four replicate food chains and all species combinations (for each level of food chain length), showed modal NR values to increase by between 1.2‰ and 2.7‰ with each additional trophic level (Fig. 3). Overlap in 95% credible intervals between different food chain lengths (Fig. 3a) was reduced when wheat plant combinations were excluded (Fig. 3b), and then further reduced when maize plant combinations were also excluded (Fig. 3c). Notably, there was no subsequent change in 95% credible intervals when nettle plants were additionally removed (Fig. 3d), suggesting that these wild plants did not contribute noticeably to variation in estimates of NR. Following removal of wheat and maize plant combinations, increase in modal NR with trophic level was more closely matched at 1.7‰ and 2.4‰. Removal of nettle plant combinations increased modal values, most likely due to nettle-aphid discrimination factors being lower than some other trophic links (Table 1).

### How does carbon range change with food chain length?

**Figure 4:**
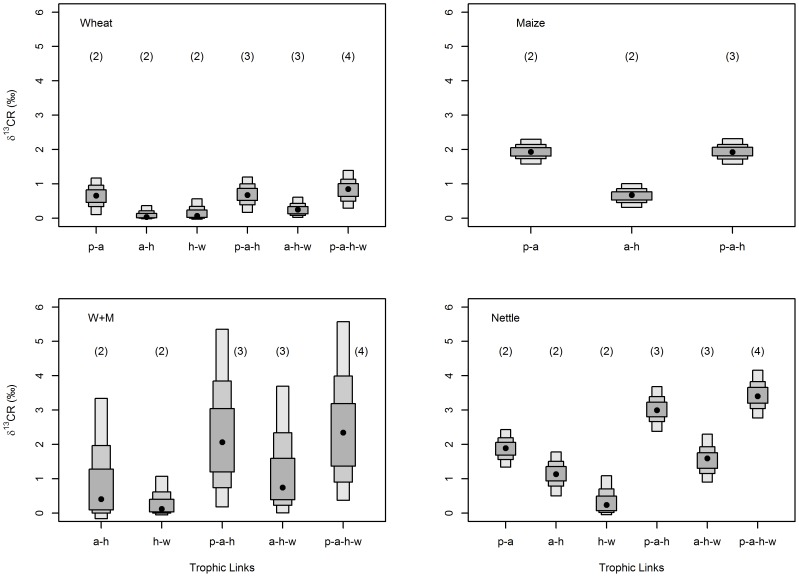
Probability distributions of mean carbon range (CR) for different food chain lengths within each food chain. Distributions based on resampling (*n* = 10,000) of uncertainty in sample mean estimates. 50%, 75% and 95% credible intervals as in Fig. 2. x-axis: p = plant, a = aphid, h = hoverfly, w = wasp. Numbers in parenthesis show food chain length.

For estimates of carbon range (CR), we observed few consistent patterns in CR across differing food chain lengths within or among food chains (Fig. 4). Wheat and maize food chains both showed no pattern of change in CR with changing food chain length; wheat CR was <1‰ across two, three and four trophic levels, while maize CR was 2‰ for combinations of both two and three trophic levels. For the w+m food chain there was marginal increase in modal CR with food chain length (two to four trophic levels ≈0.3‰ to 2.3‰) but this was considerably less than variation in mean estimates as shown by large 95% credible intervals (>3‰), on account of two isotopically disparate δ^13^C plant sources. Conversely, the wild nettle food chain trended towards greater modal CR with food chain length (two to four trophic levels ≈1‰ to 3.5‰); however, overlap in 95% credible intervals between different food chain lengths was observed, while modal values of different species combinations of the same food chain length also differed by ≈1‰ to 1.5‰.

## Discussion

We tested the accuracy of isotopic measures of trophic structure against known trophic positions using well replicated terrestrial plant and invertebrate food chains spanning four trophic levels. We found that despite some overlap in nitrogen range (NR) between longer food chains, across a range of different species combinations and food chains NR generally quantified food chain length well, suggesting robustness to observed variation in discrimination. Additionally, we found few consistent trends in δ^13^C discrimination with typically small (0.6‰ to 1.9‰) or no enrichment, and concurrently little and inconsistent change in CR with food chain length, emphasising the utility of δ^13^C to trace diet–consumer pathways. We suggest our estimates of food chain trophic structure are particularly robust because they were calculated using resampling procedures, allowing for propagation of variance in Δδ^15^N and Δδ^13^C into quantified uncertainty in final structural estimates.

### How consistent are Δδ^15^N and Δδ^13^C between trophic levels and among food chains?

#### δ^15^N

Within species variation in δ^15^N of 0.3‰ to 0.9‰ (excluding plants) was observed, and was a likely consequence of differences in Δδ^15^N between individuals due to compositional differences in food consumed and metabolic differences in assimilation of that food [13], [27], [36] (and references therein). An additional anomalous observation was large δ^15^N variation in wheat and maize plants that was not observed in wild nettle plants, suggesting that laboratory conditions affected variation. As such variation was not observed for δ^13^C which is sourced from the atmosphere and showed much smaller comparable variation across wheat, maize and nettle plants, we speculate δ^15^N variation was explained by micronutrient pockets in laboratory soil medium through incomplete homogenisation. Alternately, stress, as caused by unnaturally high and variable laboratory aphid density, may have affected plant metabolism and hence nitrogen balance, as shown for other taxa [37], [38]. As we pooled aphids to produce each aphid sample, plant δ^15^N variation likely averaged across these multiple aphids, explaining why large δ^15^N variation was not subsequently seen in aphids.

We found significant δ^15^N enrichment from source to consumer in a majority of instances, varying between 1.4‰ and 3.3‰, a range concurrent with literature estimates [11]–[13]. Averaged across food chains, such enrichment was 2.2‰ (excluding discrimination from wheat and maize plants to their aphids). This value is marginally lower than literature average estimates for invertebrates of 2.5‰ [12], [13], and lower than the overall literature estimate of 3.4‰ which is commonly employed to calculate trophic levels and food chains (e.g. [11], [23], [24]). This higher overall literature value may be reflective of trophic discrimination factors associated with vertebrates [13] and thus the lower enrichments noted in our insects re-emphasises the need to use taxa-specific discrimination factors when using isotopic data to calculate ecological parameters [13]. Additionally, broad enrichment range of 1.9‰ (1.4‰ to 3.3‰) in our results, as concurrent with previous studies [11]–[13], demonstrates variation in trophic discrimination factors is an important artefact in isotopic data that should be accounted for. Thus the use of averaging to produce commonly used trophic discrimination factors excludes variation in discrimination from final ecological estimates. Given such variation was present in our system, our subsequent use of resampling procedures allowed us to propagate discrimination uncertainty into our estimates of food chain structure, producing estimates that we contend are more accurate and hence ecologically robust [20].

In this study, as a single exception in source to consumer enrichment, wheat feeding aphids were on average depleted in δ^15^N relative to hosts, concurring with other studies [32], [39]. A previously observed negative relationship between aphid δ^15^N discrimination and host plant total nitrogen content [32] is consistent with our results; aphid enrichment was observed on maize and nettle plants that had low total nitrogen contents of 1.7% and 2.7% respectively, while wheat plants had higher nitrogen of 5.4%.

While examining sources of variation in Δδ^15^N, we found a significant interaction between trophic link and food chain type, suggesting effects of these variables were inconsistent and thus could not be generalised. This result is in close agreement with recent observations of discrimination variation at broad spatial scales in wild systems whereby variation was dissimilar across taxa within and between trophic groupings [28]. Mechanistic underpinnings explaining variation in Δδ^15^N are beyond the scope of this study but briefly, for different trophic links and for the same trophic links on different food chains, variation in Δδ^15^N was likely due to differences in nitrogen [32] and protein [40] quality of food, and/or consumer's differing metabolic assimilation of that food [27] (and references therein).

#### δ^13^C

Trophic discrimination of δ^13^C from source to consumer was inconsistent between trophic links and food chain type, ranging from −0.7‰ to 1.9‰, and more generally observed as either showing no change or significant enrichment of between 0.6‰ to 1.9‰, concurring with discrimination reviews [11], [13]. Given that in our system the carbon axis was broad, such inconsistent and generally small trophic discriminations meant δ^13^C was diagnostic of food chain type. Similarly, δ^13^C signatures of hoverfly predators on the w+m food chain were intermediate of their two disparate aphid sources, illustrating well the usefulness of δ^13^C data to integrate and reflect dietary sources.

### Does nitrogen range accurately determine food chain length?

Using resampling procedures to calculate nitrogen range (NR), we tested how NR changed with known food chain length within food chains, and then across food chains types. Excluding wheat plant–aphid combinations, we found that NR accurately determined food chain length within all food chains. This finding is concurrent with a previous validation showing positive correlations between trophic positions of freshwater fish estimated by both δ^15^N and traditional gut content analysis [14].

In our study, the inclusion of wheat plant–aphid combinations depressed NR measures on the wheat and w+m food chain types. This was because aphids were depleted relative to wheat plants, such that aphids were effectively base of the δ^15^N food chain while wheat constituted an additional trophic level that did not act to extend NR. This importantly shows exceptional species-specific effects may adversely affect the accuracy of isotopic measures of food chain length. As previously we had identified the wheat plant–aphid relationship as an exception to the generic enrichment in δ^15^N from source to consumer, we feel justified in concluding that, more generally, NR predicted food chain length.

Overall, when all trophic combinations for all food chain types were combined, modal NR values increased by between 1.2‰ and 2.7‰ with each trophic level (or 1.7‰ and 2.4‰ excluding wheat and maize plant combinations), suggesting a robustness of this technique for calculating food chain length. Excluding combinations including wheat and maize plants (on account of large variation in their δ^15^N signatures), 95% credible intervals of NR estimates showed some overlap between food chain lengths of three and four trophic levels, inhibiting accurate estimation of food chain length at these points of overlap. Similarly, for wild-sampled freshwater algae, invertebrate and fish food chains it has been shown that differences in δ^15^N between trophic levels was largely reflective of food chain length, though with variation in Δδ^15^N causing some uncertainty/error in estimating trophic level [28]. Current practice uses NR to estimate food chain length and determine subsequent conclusions, but rarely has NR's use been tested, with terrestrial systems particularly understudied [27]. Based on our results, we urge caution interpreting food chain length when NR values fall in known overlap boundaries; i.e. in our study NR values of 5‰ or 6‰ could be either three or four trophic levels. Such overlap was a consequence of variation in NR values within each trophic link and between trophic links of the same food chain length, both within and between food chain types, as ultimately caused by variation in Δδ^15^N within and between source-consumer pairings (Table 1). Of the few studies that have examined effects of variation in baseline δ^15^N [17] and Δδ^15^N [11], [25], [28] on error of trophic level or food chain length estimates, all have found error concurrent to the variation we show in estimates of NR. Importantly, our research diversifies these studies by testing empirical rather than mainly theoretical measures of food chains, in a controlled laboratory context, using understudied terrestrial and invertebrate systems, in addition to providing robust estimates of NR based on resampling of variation.

Additionally, as different food chain types in our experiment differed in their NR values for given food chain lengths as a consequence of variation in Δδ^15^N, so it is worth urging some caution when using NR for direct comparisons between systems of simple single-pathway food chains. It has been reasonably suggested that such variation may average out over multiple trophic levels or larger sample sizes [27], though based on our results we would call for research to further test the importance of such variation. Pragmatically, as in our study the effect of food chain type on Δδ^15^N was variable, then studies sampling across multiple food chains will absorb such variation as ‘noise’ that may be acceptable or specifically filtered out if food chain identity was categorised in analyses as a random effect. We speculate that such variation may also be averaged out when considering overall structure of larger food webs containing multiple food chain pathways.

Our overall estimates of NR were based on exact trophic positions in well replicated food chains and used resampling procedures that propagated uncertainty in discrimination factors to produce estimates that represented a full range of possible NR values. Given such an approach is likely to produce robust estimates of NR, and that more generally these estimates suggested that NR can accurately discriminate different food chain lengths, we conclude NR is a useful isotopic metric for quantifying food chain length.

### How does carbon range change with food chain length?

Using resampling procedures we calculated carbon range (CR) values for all food chain length combinations on all four food chain types. Overall, our results show that across trophic levels CR changed little and inconsistently both within and between food chain types (Fig. 4). Little change in CR over four trophic levels (modal range across food chain types <1‰ to 3.5‰; Fig.4) suggests fidelity of δ^13^C values between primary producers and top predators, as concurrent with our earlier findings of small and inconsistent δ^13^C discrimination (Table 1). Given global estimates of aquatic food chains approximate 3.5 to 4.0 trophic levels [17] little change in CR over four trophic levels in our study demonstrates more broadly the utility of δ^13^C to trace energy pathways, be they either simple singular chains or potentially when embedded within larger trophic structures. However, a potential caveat of using δ^13^C is that source signatures may need to be distinct in order to determine different energy pathways; we acknowledge that our δ^13^C axis was broad (≈17‰) with different plant species showing separation in δ^13^C values. In systems where multiple consumers feed on a single basal source, or basal sources have closely aligned δ^13^C, identification of different energy pathways may not be possible.

Stable isotope approaches to quantifying food web structure continue to proliferate in ecological studies and so improving our understanding of the accuracy and limitations of these techniques is of importance. In this study, source-consumer δ^15^N discrimination generally showed enrichment, with broad variation (range 1.9‰) between trophic levels and among food chains which propagated variation to subsequent estimates of NR. However, across a range of species combinations and food chains we show NR proved robust to such variation, distinguishing food chain length well, though some overlap between longer food chains importantly establishes limits in NR's precision. CR changed little with food chain length and hence δ^13^C is potentially a useful tracer of source-consumer interactions. Having established that variation in discrimination affected estimates of trophic structure, we recommend the use of resampling procedures to propagate variation as quantified uncertainty in final estimates of structure. Such procedures are necessary to improve accuracy and robustness of ecological conclusions in future isotopic studies. Given global estimates of aquatic food chains approximate four trophic levels, and that most food chains include invertebrates, our use of four trophic level plant-invertebrate food chains makes our findings relevant to a majority of ecological systems and contexts.

## Supporting Information

Dataset S1 **δ^15^N and δ^13^C values for all plant and invertebrates samples for all four food chains used in this research.** All invertebrate samples underwent lipid extraction prior to stable isotope analysis and thus δ^15^N and δ^13^C values given are post lipid extraction.
(XLSX)
